# Deregulation of a *Cis*-Acting lncRNA in Non-small Cell Lung Cancer May Control HMGA1 Expression

**DOI:** 10.3389/fgene.2020.615378

**Published:** 2021-01-11

**Authors:** Greg L. Stewart, Adam P. Sage, Katey S. S. Enfield, Erin A. Marshall, David E. Cohn, Wan L. Lam

**Affiliations:** Department of Integrative Oncology, BC Cancer Research Centre, Vancouver, BC, Canada

**Keywords:** long non-coding RNA, HMGA1, gene regulation, *cis*-acting, non-coding RNA, lung cancer LncRNA-Mediated Control of HMGA1 Expression

## Abstract

**Background:**

Long non-coding RNAs (lncRNAs) have long been implicated in cancer-associated phenotypes. Recently, a class of lncRNAs, known as *cis*-acting, have been shown to regulate the expression of neighboring protein-coding genes and may represent undiscovered therapeutic action points. The chromatin architecture modification gene *HMGA1* has recently been described to be aberrantly expressed in lung adenocarcinoma (LUAD). However, the mechanisms mediating the expression of *HMGA1* in LUAD remain unknown. Here we investigate the deregulation of a putative *cis*-acting lncRNA in LUAD, and its effect on the oncogene *HMGA1*.

**Methods:**

LncRNA expression was determined from RNA-sequencing data of tumor and matched non-malignant tissues from 36 LUAD patients. Transcripts with significantly deregulated expression were identified and validated in a secondary LUAD RNA-seq dataset (TCGA). SiRNA-mediated knockdown of a candidate *cis*-acting lncRNA was performed in BEAS-2B cells. Quantitative real-time PCR was used to observe the effects of lncRNA knockdown on the expression of HMGA1.

**Results:**

We identified the lncRNA RP11.513I15.6, which we refer to as HMGA1-*lnc*, neighboring *HMGA1* to be significantly downregulated in both LUAD cohorts. Conversely, we found *HMGA1* significantly overexpressed in LUAD and anticorrelated with HMGA1-*lnc*. *In vitro* experiments demonstrated siRNA-mediated inhibition of HMGA1-*lnc* in immortalized non-malignant lung epithelial cells resulted in a significant increase in *HMGA1* gene expression.

**Conclusion:**

Our results suggest that HMGA1-*lnc* is a novel *cis*-acting lncRNA that negatively regulates *HMGA1* gene expression in lung cells. Further characterization of this regulatory mechanism may advance our understanding of the maintenance of lung cancer phenotypes and uncover a novel therapeutic intervention point for tumors driven by *HMGA1*.

## Introduction

Long non-coding RNAs (lncRNAs) are a previously-underappreciated class of transcripts with a wide variety of now-recognized functions in gene regulation. Since their functional roles have been uncovered, numerous lncRNAs have been implicated in the onset of many cancer-associated phenotypes, such as progression, tumorigenesis, and metastasis (Gibb et al., 2011; Schmitt and Chang, 2016). Further, as RNA represents the functional unit for lncRNAs, rather than an intermediate as is the case with mRNAs, these transcripts are promising targets for the development of future RNA-based therapies (Amodio et al., 2018). Despite these recognized roles in tumor development, a key challenge in the translational utility of lncRNA-based research is the effective identification of their downstream target genes. In order to harness their potential for disease-specific markers and potential therapeutic targets, a better understanding of lncRNA mechanisms of action in disease is required.

An emerging class of lncRNAs – *cis*-acting – has been shown to regulate the expression of neighboring protein-coding genes, frequently including protein-coding genes with oncogenic or tumor-suppressive functions. Through a variety of mechanisms primarily occurring in the nucleus, these *cis-*acting non-coding transcripts can activate or repress transcription of neighboring genes. For example, the lncRNA *ANRIL* (antisense non-coding RNA in the *INK4* locus) interacts with polycomb repression complex 2 (PRC2), recruiting the complex to condense active chromatin and silence the genes around its transcriptional loci, including the well-known tumor suppressor INK4B (p15) (Yap et al., 2010).

Thus, *cis*-acting lncRNAs may represent novel mechanisms of cancer-gene regulation as well as potentially actionable intervention points in known cancer-driving pathways. Despite the prevalence of genetic and epigenetic deregulation events in lung adenocarcinoma (LUAD), the extent and consequences of aberrant *cis*-acting lncRNA expression on known cancer-driving genes is unknown.

High mobility group A1 (HMGA1) is part of a family of proteins involved in maintenance of chromatin architecture within the nucleus and as such, are implicated in tumorigenesis (Sgarra et al., 2018). Specifically, HMGA1 regulates a wide variety of genes through direct interactions with target sequences in promoter and enhancer regions. These downstream regulatory functions have been shown to lead to tumor development, particularly in breast cancer where the HMGA family has been shown to contribute, in part, to nearly every hallmark of cancer (Sgarra et al., 2018) (Hanahan and Weinberg, 2011). HMGA1 is enriched in several aggressive cancer types, including non-small cell lung cancer (NSCLC), where both mRNA and protein expression are substantially increased (Zhang et al., 2015). Further, overexpression of the *HMGA1* gene has been shown to be a key factor driving lung metastasis (Lin and Peng, 2016; Fu et al., 2018). In LUAD, high *HMGA1* gene expression has been associated with poor overall survival and chemotherapy resistance (Zhang et al., 2015). The oncogenic role of HMGA proteins stems from chromatin-mediated activation of cancer-driving genes such as *E2F1*, *AP1*, and *CCNA1*, as well as the repression of tumor suppressive genes such as *TP53* (Fusco and Fedele, 2007). Interestingly, overexpression of HMGA proteins has also been shown to alter the expression of non-coding genes, such as miRNAs, leading to lung development through dysregulation of the cell cycle (Pallante et al., 2015). However, the role of lncRNAs in HMGA1 regulation has not been investigated. As such we hypothesized lncRNA expression changes may be a mechanism of *HMGA1* deregulation in NSCLC. Our search for regulatory lncRNA has led to the identification of HMGA1-*lnc*, a *cis*-acting lncRNA neighboring the *HMGA1* gene at *6p21.31* that acts as alternative mechanisms of *HMGA1* deregulation.

## Results

### Cis-acting LncRNAs are deregulated in LUAD

We first sought to identify *cis-*acting lncRNAs with potential biological relevance in LUAD. We hypothesized that a biologically relevant *cis*-acting lncRNA would (i) be significantly deregulated in two LUAD datasets (Fold Change, FC: 1.5), (ii) would overlap or closely neighbor (within 1.5 Kb) protein coding genes, that (iii) were also deregulated in LUAD.

Putative *cis*-acting lncRNAs that fit these parameters were identified, and literature searches on PubMed were performed to identify neighboring protein coding genes with experimental evidence of roles in cancer biology (Supplementary Table 1). This gave us a list of deregulated putative *cis*-acting lncRNAs that may function to regulate known cancer driving genes. For example, we find a lncRNA RP11-122M14.1 neighboring protein-coding gene *NEK2*. *NEK2* expression is associated with poor survival and inhibition results in anticancer effects in many cancer types, including lung cancer (Xia et al., 2015; Yao et al., 2019).

**FIGURE 1:**
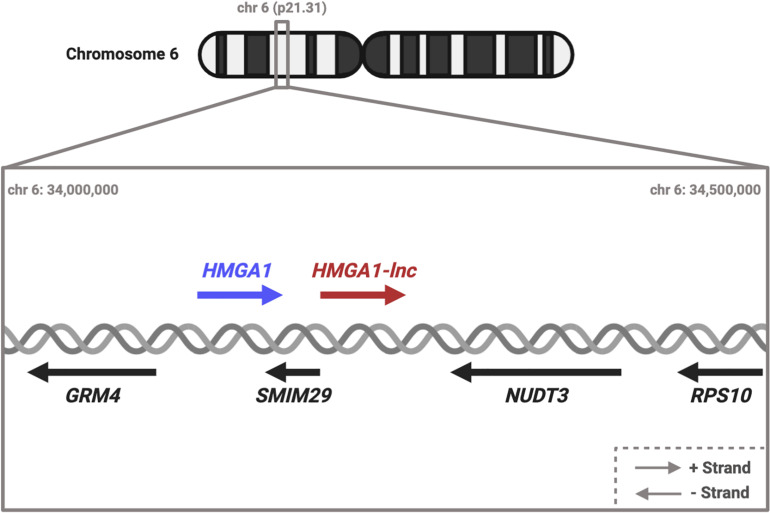
Diagram of HMGA1 transcriptional locus. HMGA1 and HMGA1-lnc are neighbors on the + strand. Located on the opposite strand are genes SMIM29, NUDT3, GRM4, and RPS10. #Dereg: Deregulation.

While many of these putative *cis*-acting lncRNAs are interesting, we decided to focus on *cis*-lncRNA-gene pairs that were deregulated in the opposite direction (discordant). We took this approach to avoid the false positives that can occur from regional “passenger” effects such as non-specific DNA copy number changes affecting multiple neighboring genes. Specifically, we decided to focus on a particular deregulated lncRNA, *RP11.513I15.6*, because of: (i) proximity to the known oncogene *HMGA1*, and (ii) the discordant expression relationship between the lncRNA and neighboring protein-coding gene. For simplicity we refer to this lncRNA as *HMGA1-lnc* (Figure 1).

### Expression of HMGA1-lnc and HMGA1 are deregulated in LUAD

**FIGURE 2:**
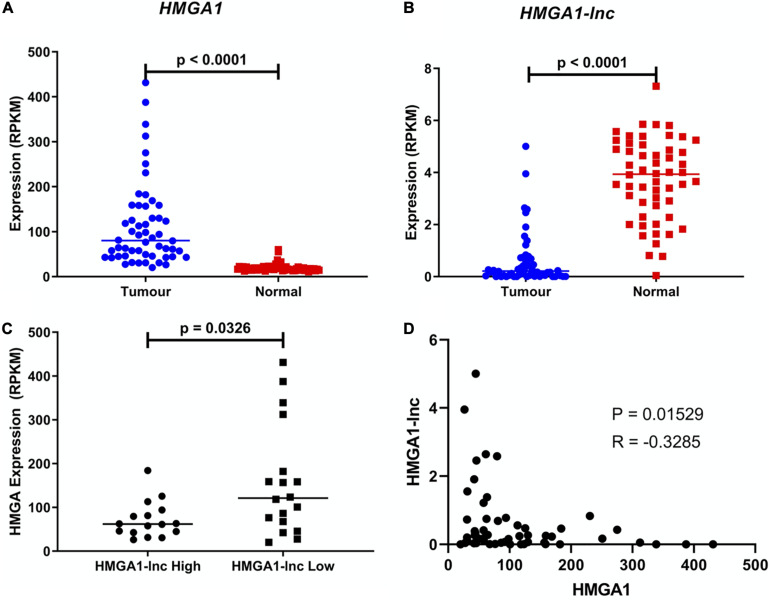
Expression of *HMGA1* and *HMGA1-lnc* in LUAD. Expression of *HMGA1* is upregulated in LUAD compared to adjacent non-malignant tissue (Students *T*-test) **(A)** while conversely, expression of *HMGA1-lnc* is downregulated in tumors (Students *T*-test) **(B)**. Additionally, tumors with high levels of *HMGA1-lnc*, have significantly lower levels of *HMGA1*, when compared to tumors with low levels of the lncRNA (Mann-Whitney *U*-test) **(C)**, and expression of HMGA1 and HMGA1-lnc are negatively correlated (Spearman’s Correlation) **(D)**. All data shown above is from the TCGA cohort (*n* = 108, 54 paired samples).

We observed the lncRNA *HMGA1-lnc* to be significantly downregulated in tumors when compared to adjacent non-malignant tissues, which holds true in both datasets (Figure 2A and Supplementary Figure 1). In contrast, the neighboring protein-coding gene *HMGA1* was found to be significantly overexpressed in both tumor datasets relative to matched non-malignant tissue (Figure 2B and Supplementary Figure 1). As mentioned above, the discordant expression in lung tumors between these genes lends further evidence to the existence of a possible regulatory relationship rather than a genomic-locus level alteration. To highlight this difference in expression, we compared the levels of *HMGA1* between tertiles of tumors with the highest and lowest expression of *HMGA1-lnc*. Interestingly we found that levels of *HMGA1* were significantly greater (*p* = 0.0326) in the low lncRNA expressing tumors (Figures 2C,D), and that *HMGA1* and *HMGA1-lnc* were negatively correlated (*p* = 0.0153). To determine if HMGA1-lnc was affecting other genes in its transcriptional locus we performed correlation analysis between HMGA1-lnc expression and other neighboring genes. Interestingly we found SMIM29, NUDT3, and RPS10 to be significantly positively correlated with HMGA1-lnc expression in the BCCA dataset, however, we did not observe significant correlations with these genes in the TCGA dataset (Supplementary Table 2). We also tested whether genes known to regulated by HMGA1 were affected by HMGA1-lnc expression. Interestingly we found two genes known to be downregulated by HMGA1 (CAV1 and FOXP1) to be positively correlated with HMGA1-lnc expression (Supplementary Figure 2). As the expression of HMGA1 and HMGA1-lnc appears to be anti-correlated, this lncRNA may be involved in the inhibition of *HMGA1* expression in normal lung contexts.

**FIGURE 3:**
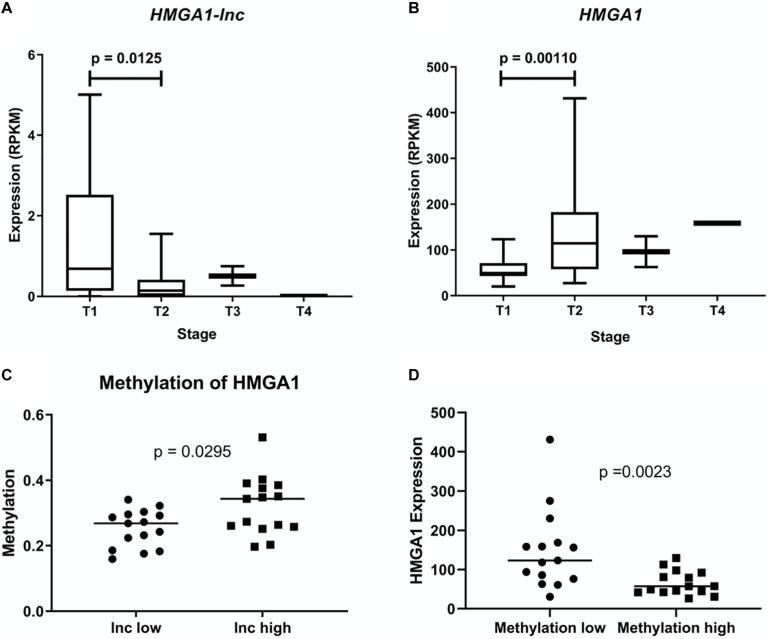
Expression of *HMGA1* and *HMGA1-lnc* is associated with tumor stage. Expression of *HMGA1-lnc* decreases with increasing tumor stage **(A)**, whereas *HMGA1* expression increases with higher grade tumors **(B)**. Tumors with high levels of HMGA1-lnc expression (Tertiles) had significantly higher levels of HMGA1 methylation than tumors with low levels of HMGA1-*lnc* **(C)**. Expression of HMGA1 is higher in tumors with low levels of HMGA1 DNA methylation compared to tumors with high levels of DNA methylation (tertiles *n* = 30) **(D)**.

As expression of *HMGA1* has been previously described to increase with tumor stage and cancer aggressiveness, we examined whether expression of *HMGA1-lnc* was inversely associated with stage (Zhang et al., 2015). As the majority of our tumor samples were Stage | and || we performed a Mann-Whitney *U*-test between these two groups in our larger dataset (TCGA) to identify significant associations (Figures 3A,B). Interestingly, while *HMGA1* was associated with increased tumor stage (*p* = 0.0011), the opposite was true for *HMGA1-lnc*, where expression of the lncRNA is significantly decreased in more advanced tumors (*p* = 0.0125). Further, as the TCGA dataset has paired DNA methylation data in the same tumors, we were able to investigate whether expression of *HMGA1-lnc* was associated with changes in DNA methylation to the *HMGA1* locus. When we compared tumors with high and low expression of *HMGA1-lnc* (tertiles), we found that tumors with high *HMGA1-lnc* had significantly higher methylation of *HMGA1* (Figure 3C). We also find that *HMGA1* expression is significantly higher in tumors with low levels of HMGA1 methylation (Figure 3D). This may indicate that *HMGA1-lnc* plays a role in regulating the DNA methylation state of *HMGA1*.

### HMGA1-lnc affects HMGA1 expression

**FIGURE 4:**
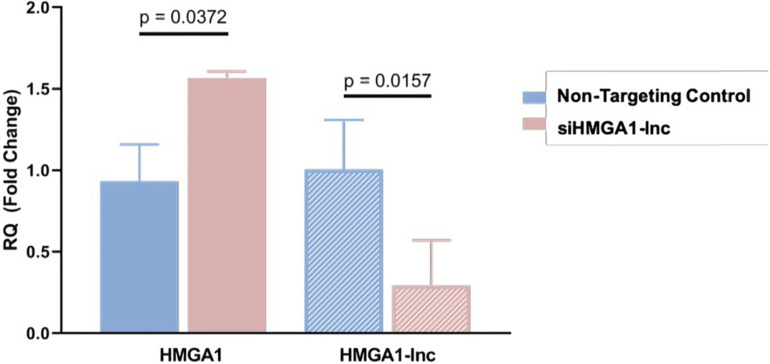
Inhibition of HMGA1-lnc results in increases of HMGA1 expression. SiRNA mediated inhibition of *HMGA1-lnc* was performed in normal bronchial epithelial cells and resulted in significant reduction of the lncRNA HMGA1-lnc. Conversely, cells where the lncRNA was inhibited showed significant increases in protein-coding *HMGA1* expression.

To determine whether loss of *HMGA1-lnc* expression is a mechanism of *HMGA1* overexpression in the lung, we performed a siRNA-mediated knockdown of *HMGA1-lnc* in the BEAS-2Bs using a pool of siRNAs specific to *HMGA1-lnc*. As HMGA1-*lnc* is downregulated in tumors, we used a non-malignant lung cell line (BEAS-2Bs) to demonstrate the effect of downregulation of *HMGA1-lnc* on *HMGA1*. We then quantified expression changes using qRT-PCR as described in Methods. From this, we observed a 3.42-fold reduction of the lncRNA after 48 h. Strikingly, in the cells with reduced *HMGA1-lnc* expression, the mRNA expression levels of *HMGA1* were increased by 1.57-fold compared with cells transfected with non-targeting control siRNAs (Figure 4). This observed increase in *HMGA1* levels in the lncRNA-inhibited cell lines, suggests that *HMGA1-lnc* may act to negatively regulate the expression of *HMGA1*, and that downregulation of this lncRNA in LUAD may be a mechanism for whereby this well-known cancer-driving gene becomes overexpressed in certain LUAD tumors.

## Discussion

The role of protein-coding genes in the onset and progression of LUAD is well-established; however, there remains a lack of treatment options for patients who do not harbor one of the few clinically-actionable driver-gene alterations. LncRNAs have been shown to have important roles in the regulation of cancer-associated genes, but complex folding patterns and unknown binding motifs make lncRNAs particularly challenging to functionally characterize. Here, we used an approach that considered the genomic location, as well as the known function of neighboring oncogenic protein-coding genes, to find and characterize a novel *cis*-acting lncRNA deregulated in cancer, which we refer to as *HMGA1-lnc*.

Further, we found that this approach could be applied to other lncRNAs, finding several deregulated lncRNAs neighboring cancer associated protein-coding genes. This included well-known lung-cancer-associated genes such as *NEK2*, suggesting that there may be a selective pressure for the deregulation of these lncRNAs in order to release these cancer-promoting genes from negative regulation. Thus, alterations in lncRNA expression may consequently disrupt coding-gene expression as a means of promoting tumor development. This may be a useful methodology for researchers to use to identify other *cis*-acting lncRNAs that may be regulating genes of interest. However, it is worth noting that using a genomics-based approach to identify these *cis*-gene relationships is not without potential pitfalls. Previous studies have shown that many *cis*-acting lncRNAs have positive expression relationships with their neighbors in several tissue types (Balbin et al., 2015). However, genes in the same vicinity are often subject to regulation that can affect whole genomic regions, such as silencing through chromatin condensation. In particular, tumors often have significantly elevated levels of these broad genomic alterations to the DNA, which enables tumor suppressor gene silencing or oncogene activation. Genes neighboring these oncogenes and tumor suppressors are often caught in these regions of alteration, and display concordant expression with these genes, a phenomenon known as the passenger effect (Sheltzer and Amon, 2011; Pon and Marra, 2015). For example, frequent DNA amplification of the *MET* oncogene occurs in 5–20% of LUAD, leading the surrounding genes to display significantly increased DNA copy number (Zack et al., 2013; Presutti et al., 2015). While it is difficult to separate *cis*-acting concordant regulatory relationships from oncogene passengers without further verifying direct interactions via *in vitro* expression modulation, genes displaying discordant expression relationships with their neighbors are less susceptible to this effect.

We sought to avoid the passenger effect pitfall of concordant regulatory relationships by focusing the majority of our analysis on discordant relationships, particularly that between *HMGA1-lnc* and *HMGA1*. We found *HMGA1-lnc* to be significantly downregulated in LUAD, where its expression level is decreased 15-fold in tumors, compared to *HMGA1* which has expression levels 5-fold greater in tumors (of the TCGA cohort). These observations in tandem with anti-correlated expression relationships within tumor samples led to our hypothesis that *HMGA1-lnc* acts to repress *HMGA1* expression in non-malignant samples. HMGA1 is known to interact with large transcriptional networks in order to drive cancers (Sumter et al., 2016). Interestingly we found that FOXP1 and CAV1, genes that are commonly repressed by HMGA1 in cancer, were positively associated with HMGA1-lnc expression (Treff et al., 2004; Schuldenfrei et al., 2011). This may indicate that HMGA1-lnc expression is able to have the opposite effect on these cancer-associated transcriptional networks (Supplementary Figure 2). Consequently, the finding that *HMGA1-lnc* was downregulated with increasing stage, while *HMGA1* expression increased with more advanced stages strengthened this putative regulatory relationship.

Methylation data for the TCGA samples allowed us to query whether expression of *HMGA1-lnc* affected the DNA methylation of *HMGA1*. Indeed, we found *HMGA-lnc* expression to be significantly correlated with DNA methylation of *HMGA1*. Further, other well-known *cis*-acting lncRNAs are known to function through regulation of the methylation state of their neighboring genes. For example, the lncRNA *TARID* which recruits GADD45A to actively demethylate the tumor suppressor TCF21 (Arab et al., 2014). This hints at one possible mechanism of *HMGA1-lnc* action, where expression may result in active methylation of *HMGA1*. We also tested the expression relationship between HMGA1-lnc and neighboring genes SMIM29, NUDT3, GRM4, and RPS10 to see if they were potentially affected by expression of this lncRNA. While we saw positive correlations for three of these genes in the BCCA dataset, these gene relationships were not consistent with the TCGA dataset (Supplementary Table 2). Further studies will be required to fully elucidate the effect of HMGA1-lnc on its neighboring genes, and the potential mechanism of its action.

To verify that *HMGA1-lnc* was able to affect *HMGA1* expression levels, we performed siRNA-mediated knockdown of the lncRNA in cells derived from normal lung epithelium (BEAS-2B cells). We chose to use an inhibitory model to best recapitulate the phenotype we seen in our RNA sequencing datasets where HMGA1-lnc is downregulated in tumor samples. Additionally, some common methods of gene modulation such as exogenous over-expression may not work well to activate *cis*-acting lncRNAs. For example, the function of some *cis*-acting lncRNAs is tied to the act of transcription, rather than the produced transcript itself (Margaritis et al., 2012; Pelechano and Steinmetz, 2013). When the lncRNA was inhibited we noted significant increases in *HMGA1* mRNA, confirming that this lncRNA directly regulates the expression of *HMGA1 in vitro*. Previous studies modulating expression levels of *HMGA1* in these same normal lung epithelial cells (BEAS-2B) have shown that increased *HMGA1* expression leads to transformed phenotypes and increases in anchorage-independent cell growth (Hillion et al., 2009). These results suggest that downregulation of this previously-uncharacterized lncRNA may lead to *HMGA1* upregulation, potentially driving the onset of these same cancer phenotypes in normal human lung epithelial cells.

Our study shows that a novel *cis*-acting lncRNA, *HMGA1-lnc*, is deregulated in LUAD, which represents an alternative mechanism of activation of the oncogene *HMGA1*. The methodology used in this work was able to identify candidate *cis*-acting lncRNAs that may regulate cancer driving genes. This approach may be useful in the study of other cancer types, where with different genetic backgrounds, other *cis*-acting lncRNAs may be regulating other known oncogenes or tumor suppressors specific to other cancer types. Interestingly, this lncRNA has been described previously in hepatocellular carcinoma (HCC), where its expression was used in an RNA-based biomarker panel used to differentiate HCC patients from patients with chronic hepatitis C virus, and healthy controls (Abd El Gwad et al., 2018). As HCC is a malignancy known to be driven by HMGA1 it would be interesting to determine if HMGA1-lnc was similarly downregulated in these tumors in order to deregulate expression of HMGA1. Further, as *HMGA1* is a known oncogene in other forms of malignancy, particularly breast cancer, it would be useful to determine if this lncRNA based mechanism is common across cancer types. If so, this interaction could represent a novel clinical intervention point in *HMGA1*-driven cancers.

## Materials and Methods

### Sample collection and processing

Collection and sequencing of both cohorts were performed in congruent manners as described in a previous publication (Stewart et al., 2019). Two separate cohorts of raw RNA sequencing reads from LUAD tumors with matched adjacent non-malignant tissues were used in this study: an in-house microdissected cohort collected at the BC Cancer Research Centre (BCCA, *n* = 72, or 36 pairs) (Supplementary Table 3), and a secondary set of LUAD tumors and matched non-malignant tissue (TCGA, *n* = 108, or 54 pairs) were downloaded from The Cancer Genome Atlas (TCGA) Data Portal^1^. The BCCA cohort was composed of fresh-frozen LUAD tumors and matched non-malignant lung parenchymal tissue collected from 36 patients at the Vancouver General Hospital. Consent obtained from the tissue donors of this study was both informed and written, and sample collection was approved from the University of British Columbia-BCCA Research Ethics Board. Matched non-malignant samples were collected from areas >2 cm away from the tumor. In order to reduce contaminating sequences derived from alternative cell types, tissue microdissection was guided by a pathologist. Samples used in this study contained >80% tumor cell or >80% non-malignant cell content. Total RNA was extracted using Trizol reagent and standard procedures.

### RNA sequencing and processing

Libraries were constructed at Canada’s Michael Smith Genome Sciences Center using total RNA extracted from tumors and matched normal tissue (GSC, Vancouver, Canada). RNA quality was assessed using the Agilent Bioanalyzer RNA nanochip, and arrayed into a 96 well plate. RNA containing poly A sequences were then purified using the MultiMACS mRNA isolation kit 2 μg total RNA with on-column DNaseI-treatment (Miltenyi Biotec, Germany). The Superscript Double-Stranded cDNA Synthesis kit was used to synthesize DS cDNA (Life Technologies, United States). Library construction was done in a paired end, strand-specific manner following the GSC library preparation protocol, and the Illumina HiSeq 2,000 platform was used for RNA sequencing. Raw sequencing reads were subject to quality control based on length (<50 nt, under two thirds of maximum read length of 75 nt) and quality level (Phred < 20 were discarded). STAR aligned (version 2.4.1d) was then used to align reads (.fastq) to the human genome (NCBI GRCh37) (Dobin et al., 2013). Quantification of aligned reads (.bam) was performed using the Ensemble Transcripts reference (Release 75) (Flicek et al., 2014). As described in a previous manuscript, raw sequencing reads for the BCCA dataset were deposited at the Bioproject http://www.ncbi.nlm.nih.gov/bioproject/516232 (Stewart et al., 2019). RNA sequencing data was downloaded for TCGA dataset from The Cancer Genome Atlas (TCGA) Data Portal^1^. Sequencing data (.bam files) was then processed as described above. Quantification of both datasets was performed using the Partek Flow platform as reads per kilobase million (RPKM).

### Gene expression analyses

To identify genes deregulated in LUAD, we performed a Wilcoxon signed-rank test on both lncRNA and protein-coding gene expression between tumor and matched non-malignant tissues. lncRNAs significantly deregulated in the same direction in both cohorts [Benjamini Hochberg-corrected *p*-value < 0.05; Fold Change (FC) > 1.5] were considered for further analyses (Supplementary Table 4). To assess potential cancer-relevant *cis-*acting lncRNAs, we identified deregulated lncRNAs neighboring protein-coding genes that were also deregulated. We then queried the literature (using PubMed) for experimental evidence of deregulated protein-coding genes with tumor biology. LncRNAs close enough to enact transcriptional or epigenetic changes (within 1.5 Kb) to protein-coding genes were considered as putative *cis*-acting lncRNAs (Supplementary Table 1; Gil and Ulitsky, 2020). To reduce the effect of passenger effects (such as DNA copy number alterations) on lncRNA:protein-coding-gene relationships, we focused on gene pairs with discordant (negatively correlated) expression patterns. Significant associations between *HMGA1-lnc* and neighboring protein-coding *HMGA1* expression were determined using a Spearman’s correlation. These results were confirmed and visualized using a Mann-Whitney *U*-Test between the upper and lower tertiles of samples based on lncRNA expression. To determine if *HMGA1-lnc* had protein coding potential we used the coding potential assessment tool (CPAT^2^) which identified the gene as non-coding (Supplementary Figure 3; Wang et al., 2013).

### Methylation analysis of HMGA1

DNA methylation data (HM450 beta-values) of *HMGA1* for the TCGA LUAD dataset was downloaded from the cBioPortal for Cancer Genomics (www.cbioportal.org). Samples were then ranked by expression of HMGA1-*lnc* and separated into high and low tertiles. The methylation of *HMGA1* was then compared between the high and low lncRNA expressing tertiles (Mann-Whitney *U*-Test). To determine if DNA methylation of HMGA1 had an effect on HMGA1 expression we compared tumors with high levels of methylation to those with low levels of methylation (tertiles). We found that tumors with low level of HMGA1 DNA methylation had significantly higher levels of HMGA1 expression (Mann-Whitney *U*-Test).

### In vitro analyses

The immortalized non-malignant epithelial lung cell line BEAS-2Bs was used to assess the effect of inhibition of the candidate lncRNA identified in the above analysis (referred to as: *HMGA1-lnc*) on *HMGA1* expression *in vitro*. Cells were cultured in serum-free medium: K-SFM supplemented with 30 μg/mL bovine pituitary extract (BPE) and 0.0002 ng/μL epidermal growth factor (EGF); maintained in an incubator at 37^*o*^C and 5% CO_2_. Once confluent, 2 mL of cell solution was seeded into each well of a 6 × 2 cm-well plate at a concentration of 50,000 cells/mL. DharmaFECT siRNAs were prepared for transfection as per manufacturer’s instructions in five conditions: (i) untreated control; (ii) a positive control siRNA targeting GAPDH (25 nM); (iii) a non-targeting control siRNA (25 nM); (iv) siRNA targeting *HMGA1-lnc* at a concentration of 12.5 nM; and (v) siRNA targeting *HMGA1-lnc* at a concentration of 25 nM (Supplementary Tables 4, 5). Non-targeting control was designed to target no known human genes and provide a baseline response for cellular exposure to siRNAs (Dharmacon, D-001210-01-D001210-05). RNA was harvested after both 48 and 72 h using the Quick-RNA^TM^ MiniPrep Kit (Zymo Research, Catalog number R1055). Total RNA was converted to cDNA using the High Capacity cDNA Reverse Transcription Kit (Applied Biosystems, Catalogue number 4374967). Gene expression was assessed using real-time quantitative PCR with custom primers specific to *HMGA1-lnc* generated by Thermo Fisher, as well as established primers for the *18S* ribosomal RNA (endogenous control), *GAPDH* (positive siRNA control), and *HMGA1*. RT-qPCR reactions were performed in triplicate as per Thermo Fisher recommended settings (denature 95°C for 15 s, anneal 60°C for 60 s, 40 PCR cycles). Relative expression was determined using the 2^–ΔΔCt^ method.

## Data Availability Statement

The datasets presented in this study can be found in online repositories. The names of the repository/repositories and accession number(s) can be found in the article/Supplementary Material.

## Ethics Statement

The studies involving human participants were reviewed and approved by University of British Columbia-BCCA Research Ethics Board. The patients/participants provided their written informed consent to participate in this study.

## Author Contributions

GS and AS were responsible for manuscript conceptualization, study design, and performance of *in silico* and *in vitro* analyses. KE, EM, and DC contributed to data collection, interpretation, and manuscript preparation. WL was the principle investigator and was involved in manuscript conceptualization, study design, and manuscript preparation. All authors contributed to the article and approved the submitted version.

## Conflict of Interest

The authors declare that the research was conducted in the absence of any commercial or financial relationships that could be construed as a potential conflict of interest.

## Footnotes

1 https://portal.gdc.cancer.gov/

2 http://lilab.research.bcm.edu/cpat/index.php
