# Supplementary material for: Deregulation of a Cis-Acting lncRNA in Non-small Cell Lung Cancer May Control HMGA1 Expression
Source: Front Genet. 2021 Jan 11;11:615378. doi: 10.3389/fgene.2020.615378 (PMC7831742; doi:10.3389/fgene.2020.615378)
Supplement: Supplementary file 2 [file Data_Sheet_2.docx]

Supplementary Material

Deregulation of a cis-acting lncRNA in non-small cell lung cancer may control HMGA1 expression

**Greg L. Stewart^1^*^†^, Adam P. Sage^1†^, Katey S. S. Enfield^1^, Erin A. Marshall^1^, David E. Cohn^1^, Wan L. Lam^1^**

^†^These authors contributed equally.

*** Correspondence:** Corresponding Author: gstewart@bccrc.ca

# Supplementary Figures and Tables

## Supplementary Figures

**A**

**B**

**C**

**Supplementary Figure 1.** **Expression of HMGA1-lnc and HMGA1 in the BCCA dataset**. Expression of *HMGA1* is upregulated in LUAD compared to adjacent non-malignant tissue (Students T-test) (A) while conversely, expression of *HMGA1-lnc* is downregulated in tumours (Students T-test) (B). Additionally, tumours with high levels of *HMGA1-lnc*, have significantly lower levels of *HMGA1*, when compared to tumours with low levels of the lncRNA (Mann-Whitney U-test) (C) All data shown is from the BCCA dataset


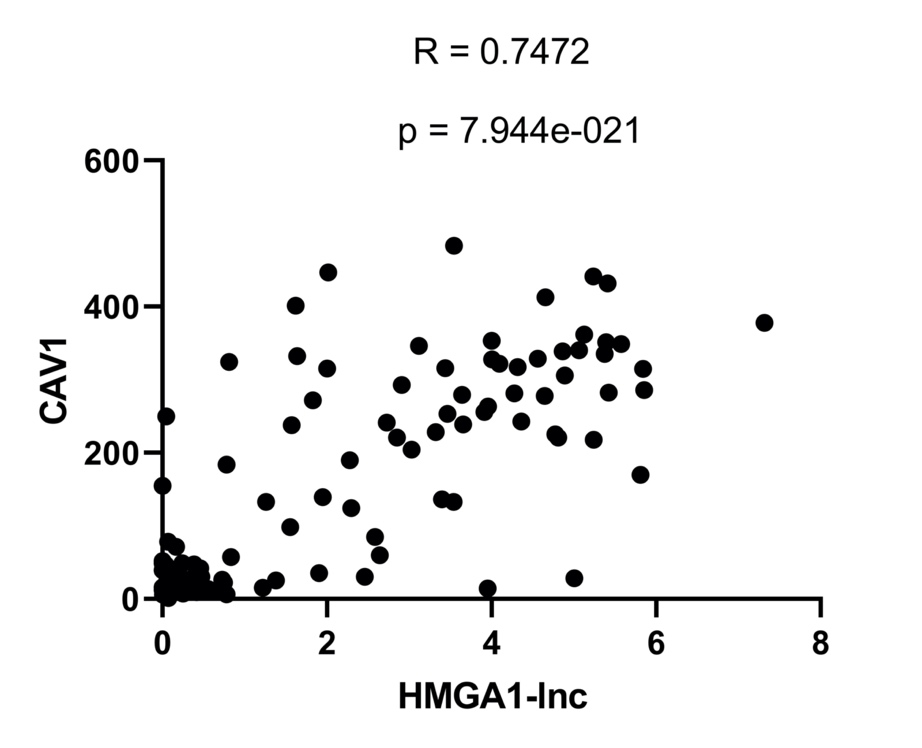

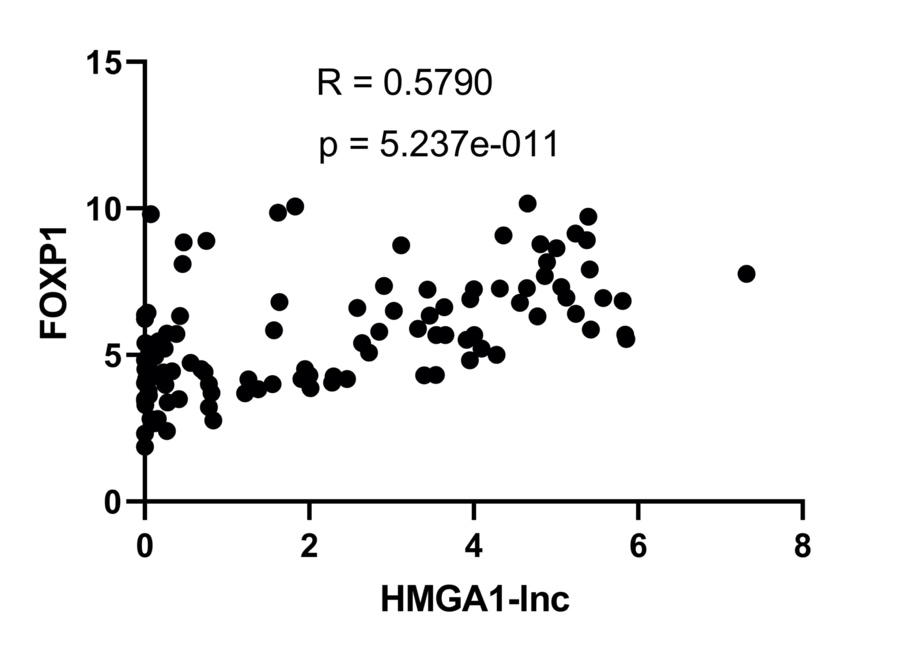


**Supplementary Figure 2. Correlation of *HMGA1-lnc* and *HMGA1* target genes**

Genes known to be repressed by HMGA1in cancer (CAV1, FOXP1) are positively correlated with HMGA1-lnc expression (Spearman’s correlation).

##
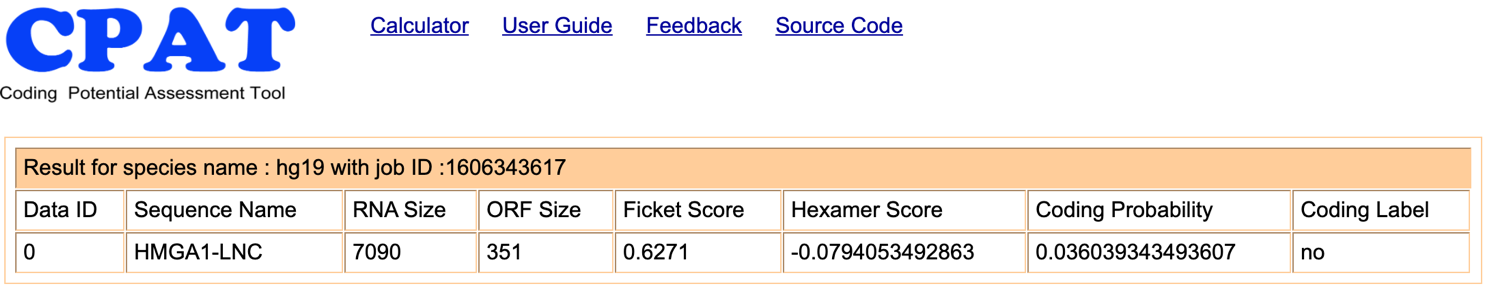


## Supplementary Figure 3. Coding potential of RP11.513I15.6 (*HMGA1-lnc*)

The full sequence of RP11.513I15.6 was run through the Coding Potential Assessment Tool to determine the potential of this transcript to code for a protein product

## Supplementary Tables

**Supplemental Table 1. Putative deregulated cis-acting lncRNAs and their neighbouring genes**

**Supplemental Table 2. Gene expression correlations with HMGA1-lnc**

**Supplemental Table 3. Clinical characteristics of Patients in the BCCA, and TCGA sample cohorts**

**Supplemental Table 4. Deregulated prospective cis-acting lncRNAs (BHC-corrected p-values).**

**Supplemental Table 5. SiRNA sequences used to target HMGA1-*lnc***

**Supplemental Table 6. qRT-PCR probes**
